# Redescription of *Ehlersileanira vulturis* (Horst, 1917) n. comb. reinst. and description of two new species of *Ehlersileanira* (Annelida, Sigalionidae) from Indonesia and the Philippines

**DOI:** 10.7717/peerj.20886

**Published:** 2026-03-16

**Authors:** Christopher Cruz-Gómez, Cheah Hoay Chuar

**Affiliations:** 1 Departamento de Sistemática y Ecología Acuática, El Colegio de la Frontera Sur, Chetumal, Quintana Roo, Mexico; 2 St. John’s Island National Marine Laboratory, Tropical Marine Science Institute, National University of Singapore, Singapore

**Keywords:** Central Indo-Pacific, Morphology, Scale worms, Siboga expedition

## Abstract

*Ehlersileanira* Pettibone, 1970 was proposed to include sigalionids (Annelida, Sigalionidae) with a median antenna with basal auricles and biarticulated style. *Sthenelais incisa Grube, 1877* described from Congo is its type species, together with a few junior synonyms, such as *Sthenelais simplex Ehlers, 1887* from the Gulf of Mexico, *Leanira vulturis* Horst, 1917 from Indonesia and *Leanira izuenzis Takahashi, 1938* from Japan. However, in a previous study, it was shown that *S. incisa*, now *Ehlersileanira incisa*, included valid species that were once considered its junior synonyms, as in the case of *S. simplex*, which is now considered distinct from *E. incisa*. Therefore, a re-examination of its junior synonyms is needed to clarify their status. Type material of *Leanira vulturis* was studied, as well as non-type specimens of the genus from the same region. The main results include the reinstatement of *L. vulturis*, which is shown to be distinct from but congeneric with *E. incisa*. *Ehlersileanira vulturis* n. comb. reinst. differs from *E. incisa* in having larger auricles and style in the median antenna, middle region segments bearing sinuous elytra, and the tubular nephridia start from segment 25; whereas *E. incisa* has smaller auricles, style, middle region elytra are uniform, and the tubular nephridia start from segment 9. Two new species of the genus from the Indo-Pacific are also described: *Ehlersileanira longistyla* n. sp. and *Ehlersileanira marinduquensis* n. sp. *Ehlersileanira longistyla* n. sp. can be distinguished from the rest of its congeners in having a long median antennal style, large inner palpal sheaths and the lowest neurochaetae unit with non-canaliculate blades. On the other hand, *E. marinduquensis* n. sp. resembles *E. vulturis*, but can be recognized by the former’s robust body shape, small style, and tentacular cirri and neurochaetal blades. A key to all *Ehlersileanira* species is also included.

## Introduction

The Siboga Expedition (1899–1900) was a major scientific expedition led by the Dutch Royal Navy to document the deep basins of the Central Indo-Pacific region, especially from the Dutch East Indies (now Indonesia) (Van Aken, 2005). The expedition’s main goal was to gather data in the following areas: zoology, botany, oceanography, and geology. During the expedition, a wide range of organisms were collected, from vertebrates such as fish to invertebrates including annelids (Weber, 1902). Among the annelids, errant polychaetes were described in a series of contributions by Horst (1903–1924) and partially addressed by Augener (H. Augener, 1920–1930, unpublished data). Augener had been studying specimens from this expedition, but his work was interrupted and remained incomplete due to a serious illness in 1937 (Pettibone, 1970a). Pettibone (1970a) later revisited Augener’s notes and descriptions, publishing his findings, which also included the description of two new genera of scale worms. In the early 21st century, further contributions to the study of errant polychaetes from the expedition were made by Hutchings & McRae (1993), focusing on Aphroditidae Malmgren, 1867, and by Aguado, San Martín & Ten Hove (2008), addressing Syllidae Grube, 1850.

Amongst the polychaetes from the Siboga expedition studied by Horst (1917), new forms of Sigalionidae Kinberg, 1856 were described, including six new species in the genus *Leanira* Kinberg, 1856. *Leanira vulturis* Horst, 1917 described from Madura Strait, Indonesia was one of them. Specimens of *L. vulturis* were recognized as distinct from the rest of *Leanira* species in having basal auricles and a short biarticulate antenna (Horst, 1917), even though these two features were previously observed in specimens originally placed in *Leanira* and *Sthenelais*. Subsequently, Pettibone (1970b) reviewed *Leanira*, transferring specimens with a median antenna with basal auricles and a biarticulate short style, among other features, to the new genus *Ehlersileanira* Pettibone, 1970. Pettibone (1970b) chose *Sthenelais incisa* Grube, 1877 as the type species of the genus. Following Augener (1918), she considered *Sthenelais simplex* Ehlers, 1887 a junior synonym; moreover, she also included among synonyms *Leanira izuensis* Takahashi, 1938 and *L. vulturis* Horst, 1917. *Ehlersileanira* remained a monospecific genus until recently, when *Ehlersileanira andamanensis* Aungtonya & Eibye-Jacobsen, 2016 was described from the Andaman Sea, and *S. simplex* from the Gulf of Mexico was evaluated, reinstated and transferred to *Ehlersileanira* (Cruz-Gómez, 2022a). However, *L. izuensis* from Japan and *L. vulturis* from Indonesia have not been evaluated since Pettibone’s revision.

Thus, *Ehlersileanira* currently contains three valid species: *E. incisa* (Grube, 1877) the type species, the recently described *E. andamanensis* Aungtonya & Eibye-Jacobsen, 2016, and *E. simplex* (Ehlers, 1887) (Read & Fauchald, 2024). Only *E. incisa* and *E. andamanensis* have records in the Central Indo-Pacific (Horst, 1917; Pettibone, 1970b; Aungtonya & Eibye-Jacobsen, 2016). It should be noted that records of *E. incisa* in the region were made as *L. vulturis*. Nonetheless, the original description of *L. vulturis* by Horst (1917) included features that agree with *Ehlersileanira* (as indicated above) but its morphology does not entirely agree with the type species of the genus, *E. incisa*. Thus, the validity of this combination should be re-evaluated (see also Aungtonya & Eibye-Jacobsen, 2016; Cruz-Gómez, 2022a). Here, we evaluate the sigalionids of the genus *Ehlersileanira* from the Central Indo-Pacific, redescribe *L. vulturis*, and describe two new species.

## Materials and Methods

The morphology was analyzed in type and non-type specimens. Measurements of total length and width from the anterior end to segment 30 were taken. One specimen of the syntype series of *L. vulturis* was already dissected (**ZMA 529.2**); therefore, new dissections were not required. Type specimens of the new species included the dissection of the first three right segments and three right segments from the middle section (segments 20–30). The first three right elytra and the right middle and posterior elytra were dissected; if a right elytron was missing, the left one was dissected. Examinations were carried out using a stereo and compound microscopes. Due to the lack of contrast in the soft tissue, the specimens were temporally stained with Shirlastain®-A (SDL ATLAS) and photographed, after removal of surplus stain. Photographs were taken with a Canon T8i camera mounted on a microscope adapter. A series of pictures of each structure was digitally stacked and edited with the software Helicon Focus ver. 8. Hand drawings were made using a camera lucida and digitalized using a drawing tablet (Wacom Intuos Draw; Wacom, Saitama, Japan) displayed in the software Photoshop CC. Anterior section and one middle section parapodia of a paratype of *Ehlersileanira longistyla* n. sp. were prepared for scanning electron microscopy (SEM). These structures were dehydrated in a graded series of ethanol concentrations and hexamethyldisilazane (HMDS), and left overnight. Finally, the structures were mounted in aluminum stubs and coated with gold for observation using a JEOL-JSM-601Plus-LA scanning electron microscope at the Scanning Electron Microscopy Laboratory (LMEB), ECOSUR, Chetumal.

### Morphology

Pettibone (1970b), Aungtonya & Eibye-Jacobsen (2016) and Cruz-Gómez (2022a) described the variety of lobes in the notopodia and neuropodia; however, this method of description could be misleading and confusing. Here, the description is standardized as in other sigalionins with complex parapodia (*e.g*., *Sthenolepis* Willey, 1905). The parapodia description will only include the shape and number of lobes in noto-and neuropodia, excluding the terminology of upper or lower lobes.

The segment where branchiae first occur has been used as taxonomically relevant in classic and recent contributions (Horst, 1917; Takahashi, 1938; Pettibone, 1970b; Aungtonya & Eibye-Jacobsen, 2016); however, it has been subject to interpretation. In non-elytrogerous segments, rudimentary branchiae appear as a small tubercle or fold, and in posterior segments, the branchia is a dendritic structure that can double or triple its size. The same pattern of the structure is noted in the elytrogerous segments; therefore, we consider that the branchiae start between segments five and seven (as a small fold), but long branchiae (at least the double its size) are also included in the description of the specimens.

The description of the neurochaetae followed the terminology proposed by Cruz-Gómez (2022b), Cruz-Gómez & Blake (2024). In the descriptions, ‘×’ is used in place of ‘times’ to express the length ratio between different body features. Cruz-Gómez & Blake (2024) explained the ratio for long, medium-sized, and short blades of the genera *Sthenolepis* and *Neoleanira*; here, the blade ratio is delimited for *Ehlersileanira*. Long blades are more than 20 times as long as they are wide, medium-sized blades are between 19 and 11 times as wide, and short blades are less than 10 times as long as they are wide.

### Material examined

Type and non-type material examined, described and illustrated belong to the following institutions:

ECOSUR El Colegio de la Frontera Sur, Quintana Roo, Mexico.

MZB Museum Zoologicum Bogoriense, National Research and Innovation Agency (BRIN), Cibinong, Indonesia.

RMNH Naturalis Biodiversity Center (including Rijksmuseum voor Natuurlijke Historie, Leiden and ZMA Zoologisches Museum Universiteit van Amsterdam, The Netherlands).

USNM National Museum of Natural History, Smithsonian Institution, Washington D.C.

ZRC Zoological Reference Collection, Lee Kong Chian Natural History Museum, National University of Singapore, Singapore.

Specimens collected during the South Java Deep-Sea Biodiversity Expedition 2018 were sampled under research permit RISTEKDIKTI 80/SIP/FRP/E5/Dit.KI/III/2018 provided by the Ministry of Research, Technology and Higher Education, Republic of Indonesia.

The electronic version of this article in Portable Document Format (PDF) will represent a published work according to the International Commission on Zoological Nomenclature (ICZN), and hence the new names contained in the electronic version are effectively published under that Code from the electronic edition alone. This published work and the nomenclatural acts it contains have been registered in ZooBank, the online registration system for the ICZN. The ZooBank LSIDs (Life Science Identifiers) can be resolved and the associated information viewed through any standard web browser by appending the LSID to the prefix http://zoobank.org/. The LSID for this publication is: [lsid:zoobank.org:pub:517D778D-ED68-4DFA-A708-FB57FFDDDADF]. The online version of this work is archived and available from the following digital repositories: PeerJ, PubMed Central SCIE and CLOCKSS.

## Results

### Systematics

**Subfamily Sigalioninae Kinberg, 1856**

***Ehlersileanira* Pettibone, 1970b**

***Ehlersileanira* Pettibone, 1970b**: 19.

**Type species.** *Leanira incisa* Grube, 1877 by original designation

**Diagnosis (after Cruz-Gómez, 2022a).** Sigalioninae with prostomium dorsally fused with tentacular segment. Three antennae, with short ceratophore and short bilobed style. Ceratophore of median antenna with lateral auricles and/or ctenidia, emerging anterodorsally from prostomium; lateral antennae fused dorsally with tentacular parapodia. Dorsal tentacular crest and inner tentacular lobes absent. Nuchal organs may be visible. Inner and outer palpal sheaths present. Bulbous facial tubercle present. Segment 3 without dorsal cirri; tubercles may be present. Elytra smooth, without tubercles or papillae. Ctenidial pads present between notopodium and elytrophore, or dorsal tubercle, and ventral pedunculate ctenidial pads between segments in posterior segments. Notopodia with anterior and posterior lobes bear stylodes. Neuropodia with four neuropodial lobes, two prechaetal and two postchaetal bearing stylodes. Notochaetae spinous capillaries/simple chaetae. Neurochaetae supra-acicular simple pinnate may be present, compound spinigers with canaliculate blades.

***Ehlersileanira vulturis* (Horst, 1917) comb. nov., reinst.**

Figures 1–2

**Figure 1:**
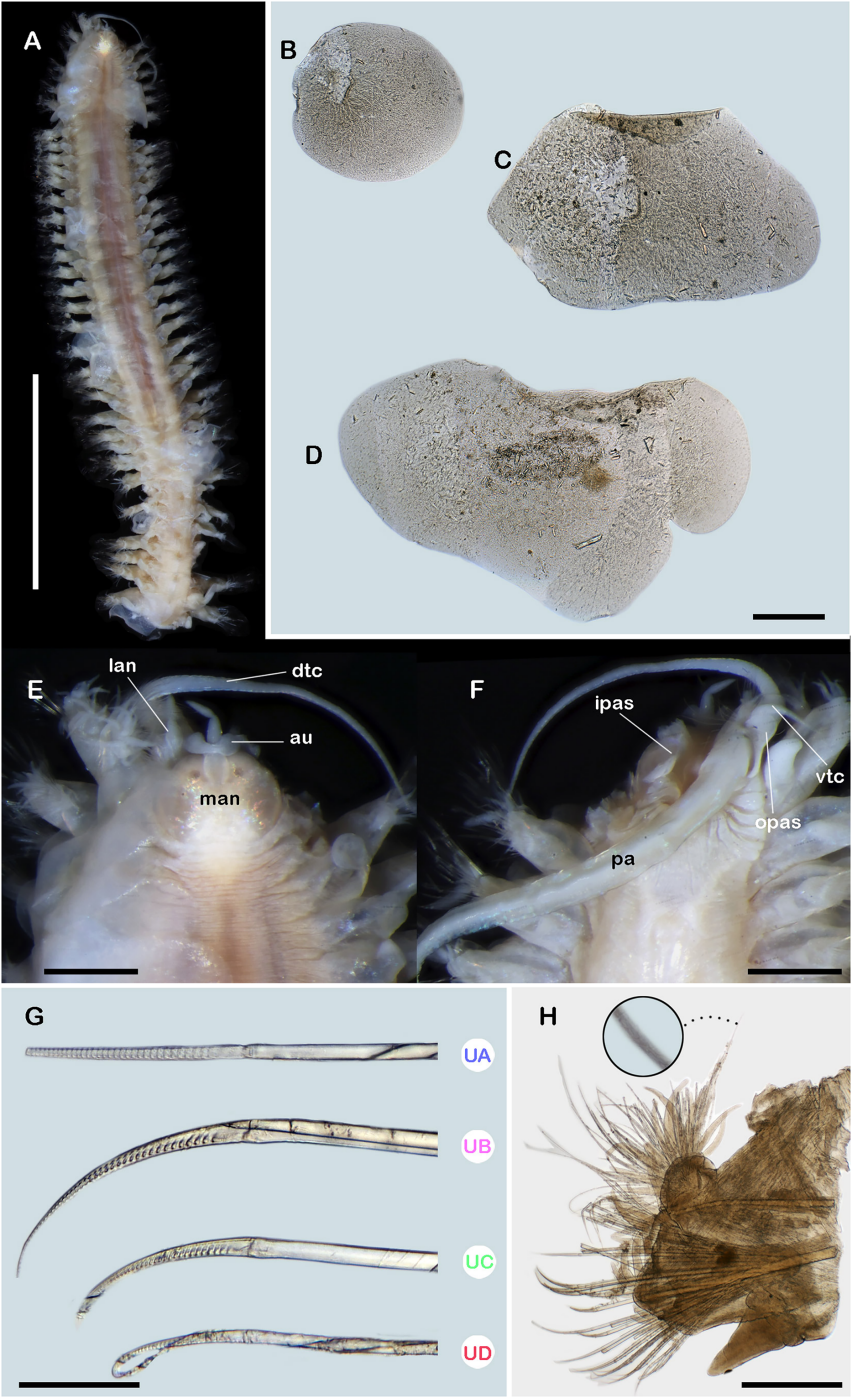
*Ehlersileanira vulturis* (Horst, 1917), holotype (ZMA 529.2). (A) Dorsal view. (B) First right elytron. (C) Second right elytron. (D) Median right elytron. (E) Anterior end, dorsal view. (F) Anterior end, ventral view. (G) Neurochaetae from segment 3. (H) Parapodium from segment 3, anterior view, inset: close-up of notochaetae. Abbreviations: au, auricles; dtc, dorsal tentacular cirri; ipas, inner palpal sheath; lan, lateral antenna; man, median antenna; opas, outer palpal sheath; pa, palp; vtc, ventral tentacular cirri; UA, upper group; UB, upper middle group; UC, middle group; UD, lowest group. Scale bars: A, 5 mm; B, E, F, 500 µm; H, 200 µm; G, 50 µm.

**Figure 2:**
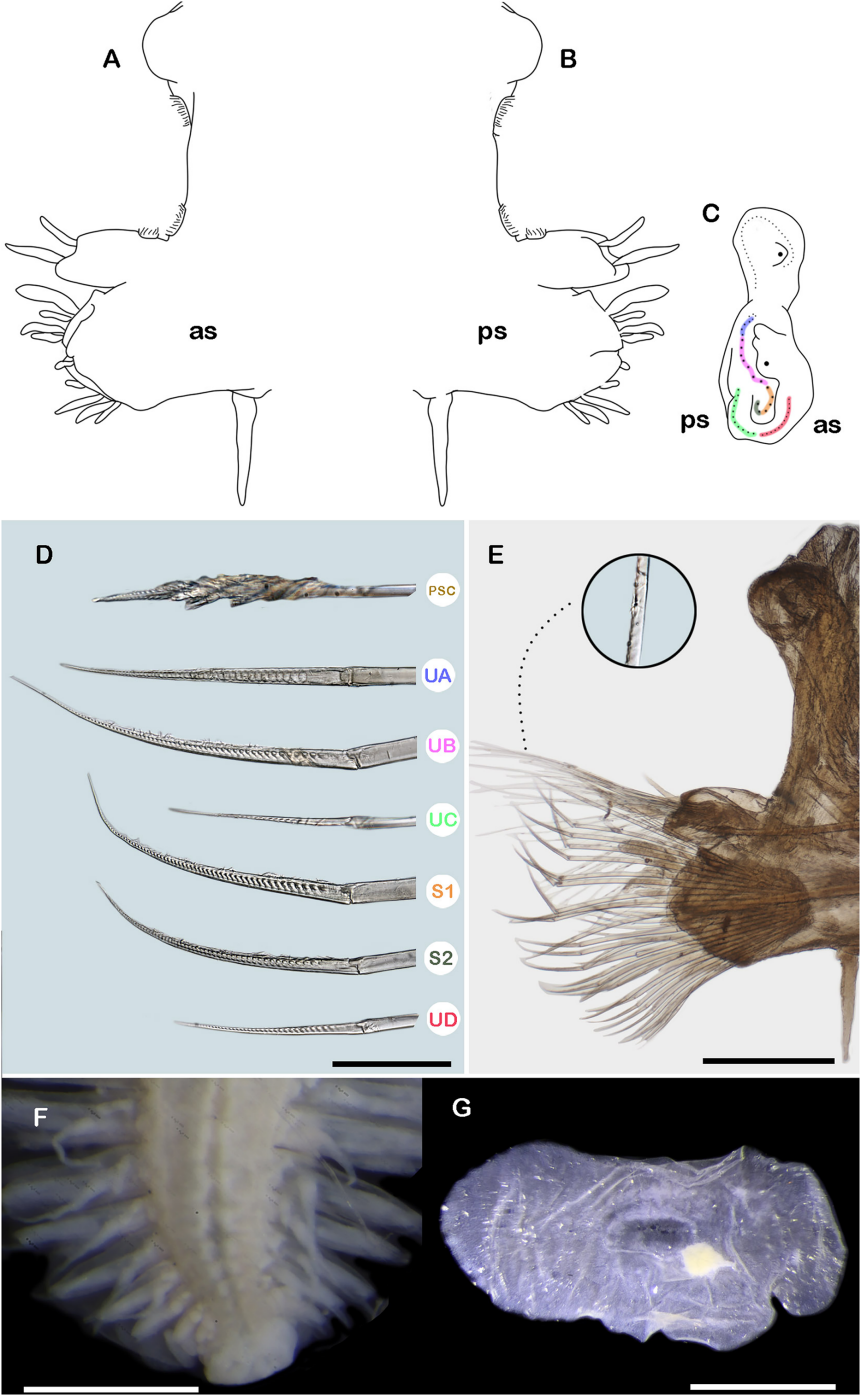
*Ehlersileanira vulturis* (Horst, 1917), holotype (ZMA 539.2). (A) Drawing of parapodium from segment ~30, anterior view. (B) Drawing of same, posterior view. (C) Diagram of same, lateral view, color lines indicate insertion of neurochaetal groups, color scheme following that shown in D. (E) Parapodium from segment ~30, anterior view, inset: close-up of notochaetae. (F) Pygidium, ventral view (RMNH.VER.1163). (G) Right posterior elytron. Abbreviations: PSC, Pinnate simple chaetae; S1, lower middle group; S2, lower group; UA, upper group; UB, upper middle group; UC, middle group; UD, lowest group; as, anterior side; ps, posterior side. Scale bars: G, 1 mm; E, F, 500 µm; D, 50 µm.

*Leanira vulturis* Horst, 1917: 118, Pl. 25, figs. 5–7 (part of *Siboga* station 47); Cruz-Gómez, 2022a: 171 (key of species referred to *Ehlersileanira*)

*Sthenolepis vulturis*.—Hartman, 1965: 14.

*Ehlersileanira incisa*.—Pettibone 1970b: 19. Syn (partim, *non* Grube, 1877).

**Type locality.** 07° 27.5′ S 113° 08.5′ E, Madura Strait, Central Indo-Pacific

**Type material. Syntypes. ZMA 529.1:** 1 spec. anterior fragment (Madura Strait, 07° 27.5′ S 113° 08.5′ E, 37 m, Sta. 1, 7 March 1899); **ZMA 529.2:** 2 specs. anterior fragments (Madura Strait, 07° 25′ S 113° 16′ E, 56 m, Sta. 2, 8 March 1899); **ZMA 529.3:** 1 spec. and fragments (Bay of Bima, 55 m, Sta. 47, 8/12 April 1899); **RMNH 1164:** 1 spec. anterior fragment (Bay of Bima, 55 m, Sta. 47, 8/12 April 1899); **RMNH 1163:** 1 spec. complete (Strait of Makassar, 03° 30′ S 116° 33′ E, 30.6 m).

**Additional material. USNM 17517:** 2 spec. (between Samar & Masbate, P.I., USS Albatross, Sta. 5,397, 245 m, 15 March 1909). **USNM17503:** 1 spec. (between Panay & Negros, P.I., Albatross sta. 5,183, 175 m, 30 March 1908). **USNM 17513:** 1 spec. (between Samar & Masbate, P.I., USS Albatross sta. 5,392, 246.8 m, 13 March 1909). **ECOSUR-3322** (1 spec. small, juvenile, 4 April 2018, 6°07.293′ S 105° 25.076′ E, 208–250 m, South Java Deep-Sea Biodiversity Expedition); **ZRC.ANN.2187** (1 spec. small, juvenile, 4 April 2018, 6° 07.293′ S 105° 25.076′ E, 208–250 m, South Java Deep-Sea Biodiversity Expedition).

**Description.** Syntype (**ZMA 529.2**) incomplete with 38 segments, 15 mm long, 2.7 mm to segment 30, 1 mm wide. Body slender, tapered, wider in the anterior section, damaged posteriorly; pale orange due to the muscular pharynx, slightly whitish posteriorly (Figs. 1A, 1E, 1F). Mid-dorsal line smooth, a few elytra remain attached, venter smooth. Elytrophores on segments 2, 4, 5, 7, then alternate segments to 25, then present in all segments. Elytrophores bulbous, slightly longer and thinner in posterior segments.

Prostomium pale orange, whitish on cerebral lobes; oval, slightly wider than long. Eyes two pairs, anterior pair directed frontally, posterior pair inserted dorsally. Lateral antennae short, inconspicuous, inserted on inner dorsal side of tentacular segment. Median antenna with ceratophore, short, ½ shorter than prostomial length; style biarticulate, distal and proximal articles subequal, ½ as long as ceratophore length; inserted on anterior margin of the prostomium. Auricles reniform, as long as ceratophore; inserted distally. Tentacular segment uniramous, chaetae verticillate, stylodes numerous, 21 dorsal lanceolate stylodes. Dorsal tentacular cirri 5–6× longer than tentacular neuropodia, ventral tentacular cirri short, slightly larger than tentacular neuropodia (Fig. 1E). Facial tubercle between palps, visible in ventral view. Palps long, reaching segment 23. Inner palpal sheaths long, outer palpal sheaths slightly shorter (Fig. 1F). Buccal cirri slightly thicker than the remaining ventral cirri. Buccal ctenidial pads, slightly enlarged, inserted anterolaterally on the buccal aperture. Ctenidial pads from segment 2; segment 2 with one dorsal ctenidial pad, succeeding segments with 2–3 ctenidial pads: two large and bulbous pads placed on dorsolateral surface of the segment; one ctenidial pad, as large as dorsolateral ones, placed on dorsal side of notopodia. Dorsal pedunculate ctenidia from segment 5, inserted between rudimentary branchiae and elytrophore. Ventral intersegmental pedunculate ctenidia from segment 19. Branchiae from segment 7, becoming larger from segment 15, 2× as long as anterior ones, filiform with cilia (Fig. 1A). Tubular nephridia from segment 25, short, slightly shorter than ventral cirri.

All elytra with smooth surfaces and margins. First right elytron small, oval (Fig. 1B). Second right elytron, oblong, margins sinuous (Fig. 1C). Median elytron larger, oblong (Fig. 1D).

Segment 3 (Fig. 1H). Notopodia rounded, slightly shorter than neuropodia; acicular lobe not differentiated with 14 dendritic stylodes. Notacicula thick, not protruding from body wall. Notochaetae with up to 40 simple verticillate chaetae, smallest as long as notopodia, longest 3× as long (Fig. 1H, inset). Neuropodia conical. Prechaetal lobe entire with three dendritic stylodes. Postchaetal lobes differentiated, divided with a deep notch, with six stylodes. Neuracicula thick, inserted in prechaetal lobe, not protruding from body wall. Neurochaetae only spinigers, handles smooth, slender (Fig. 1G). Upper group (unit A) with six chaetae, blades medium-sized, 15× as long as they are wide. Upper middle group (unit B) with four chaetae, medium-sized, 10× as long as they are wide. Middle group (unit C) with four chaetae, blades short, 9× as long as they are wide. Lowest group (unit D) with eight chaetae, blades long, 17× as long as they are wide. Ventral cirri thick, slightly shorter than neuropodia.

Segment ~30 (middle segment) (Figs. 2A, 2B, 2C, 2E). Notopodia rounded, as long as neuropodia; acicular lobe differentiated with 2 small subdistal dendritic stylodes. Notacicula thick, not protruding from body wall. Notochaetae with up to 20 simple verticillate chaetae, smallest barely longer than notopodia, longest 2× longer than notopodia (Fig. 2E, inset). Neuropodia conical. Prechaetal lobe entire, with a few small dendritic stylodes on the lateral external margin. Postchaetal lobes well differentiated with a deep notch, with a few stylodes. Neuracicula thick, inserted in prechaetal lobe, not protruding from body wall. Neurochaetae 4 pinnate simple chaetae and spinigers, handles smooth, slender (Fig. 2D). Upper group (unit A) with four chaetae, blades long, 17–18× as long as they are wide. Upper middle group (unit B) with six chaetae, blades long, 17–19× as long as they are wide. Middle group (unit C) with four chaetae, blades long, 19× as long as they are wide. Lower middle group (subunit 1) with three chaetae, blades long, 51× as long as they are wide. Lower group (subunit 2) with two chaetae, blades long, 19× as long as they are wide. Lowest group (unit D) with 8 chaetae, blades long, 19× as long as they are wide. Ventral cirri smaller than parapodia (Figs. 2A, 2B).

Posterior region lost.

**Variation.** Only one specimen with eyes present (syntype, **ZMA 529.2**), other specimens with eyes not observed. One syntype specimen (**RMNH.VER.1163)** was complete, including the pygidium. The pygidium is expanded, twice as large as the regular posterior segments. Anal cirri not observed, anus terminal (Fig. 2F).

Only one specimen with posterior elytra (**USNM 17513**). Elytra from posterior segments, larger, proximally and laterally expanded (Fig. 2G).

**Remarks.** Horst (1917) described *Leanira vulturis* based on eight specimens; however, one lot, **ZMA 529.3** includes only one specimen with three more posterior fragments, possibly from the same specimen.

Horst (1917) compared *L. vulturis* with *L. hystricis* Ehlers, 1874 and *L. tetragona* Oersted, 1845 using the features from the median antennae. The median antenna of *L. hystricis* was described as short subulate antenna (Pettibone, 1970b: 8, fig. 4a), similar to *L. vulturis* (Horst, 1917: Pl. 25, fig.7), but with the difference of lacking a pair of basal antennal auricles. On the other hand, *L. tetragona* possesses auricles in the median antenna, as in *L. vulturis*; however, the antenna is not subulate, but long and tapered (Pettibone, 1970b: 369, fig. 2a). It should be noted that the current combination of *L. tetragona* is *Neoleanira tetragona* (Pettibone, 1970c).

Specimens of *L. vulturis* differs from those of *E. incisa* in having larger auricles, expanded anteriorly, while *E. incisa* has shorter auricles, and expanded laterally (Cruz-Gómez, 2022a). Another difference located in the anterior region is the size of the style of median and lateral antennae, whereas *L. vulturis* has long style (as long as ceratophore, or longer), *E. incisa* has short styles (½ as long as ceratophore) (Pettibone, 1970b: 21, fig. 10a; Cruz-Gómez, 2022a: 167, fig. 4a). The elytra also differ between these species. Middle region elytra in *L. vulturis* have sinuous margins: anterior margin entire and uniform, lateral margin bilobed divided with a deep notch, posterior elytral lobe enlarged, whereas in *E. incisa* middle region elytra have uniforms margins: anterior margin entire and uneven, lateral margin bilobed divided by a shallow notch, posterior elytral lobe uniform (Pettibone, 1970a: 22, fig. 11i; Cruz-Gómez, 2022a: 167, fig. 4i, j).

Further, some differences are also noted after the start of some structures along the body. In *L. vulturis*, branchiae start from segment 7, ctenidial pads from segment 2, and tubular nephridia from segment 25; whereas in *E. incisa* branchiae start from segment 9, ctenidial pads from segment 7 and tubular nephridia from segment 9.

The similarities with the diagnosis of *Ehlersileanira* and the differences between *E. incisa* and *L. vulturis* support the new combination *Ehlersileanira vulturis* (Horst, 1917) comb. nov. The species is therefore regarded as distinct from *L. incisa* and is herein reinstated.

Aungtonya & Eibye-Jacobsen (2016) described *E. andamanensis* from the Andaman Sea, Thailand, and differentiated their species from *E. incisa*; however, they pointed out the morphological resemblance with *L. vulturis*, then a junior synonym of *E. incisa*. Both species share the same natural distribution in the Central Indo-Pacific (Horst, 1917; Pettibone, 1970b). However, *E. vulturis* differs from *E. andamanensis* in having only auricles in the basis of the median antenna ceratophore, a longer style in the median antenna, and elytra from the middle region with sinuous margins, whereas in *E. andamanensis* ctenidial pads are also found below the auricles in the median antennal ceratophore, a short style (as long as the ceratophore; Aungtonya & Eibye-Jacobsen, 2016: 112, fig.1ABC; 113, fig. 2A) and elytra from the middle region with rather uniform margins (Aungtonya & Eibye-Jacobsen, 2016: 113, fig 2H).

***Ehlersileanira longistyla* n. sp.**

Figures 3–5

**Figure 3:**
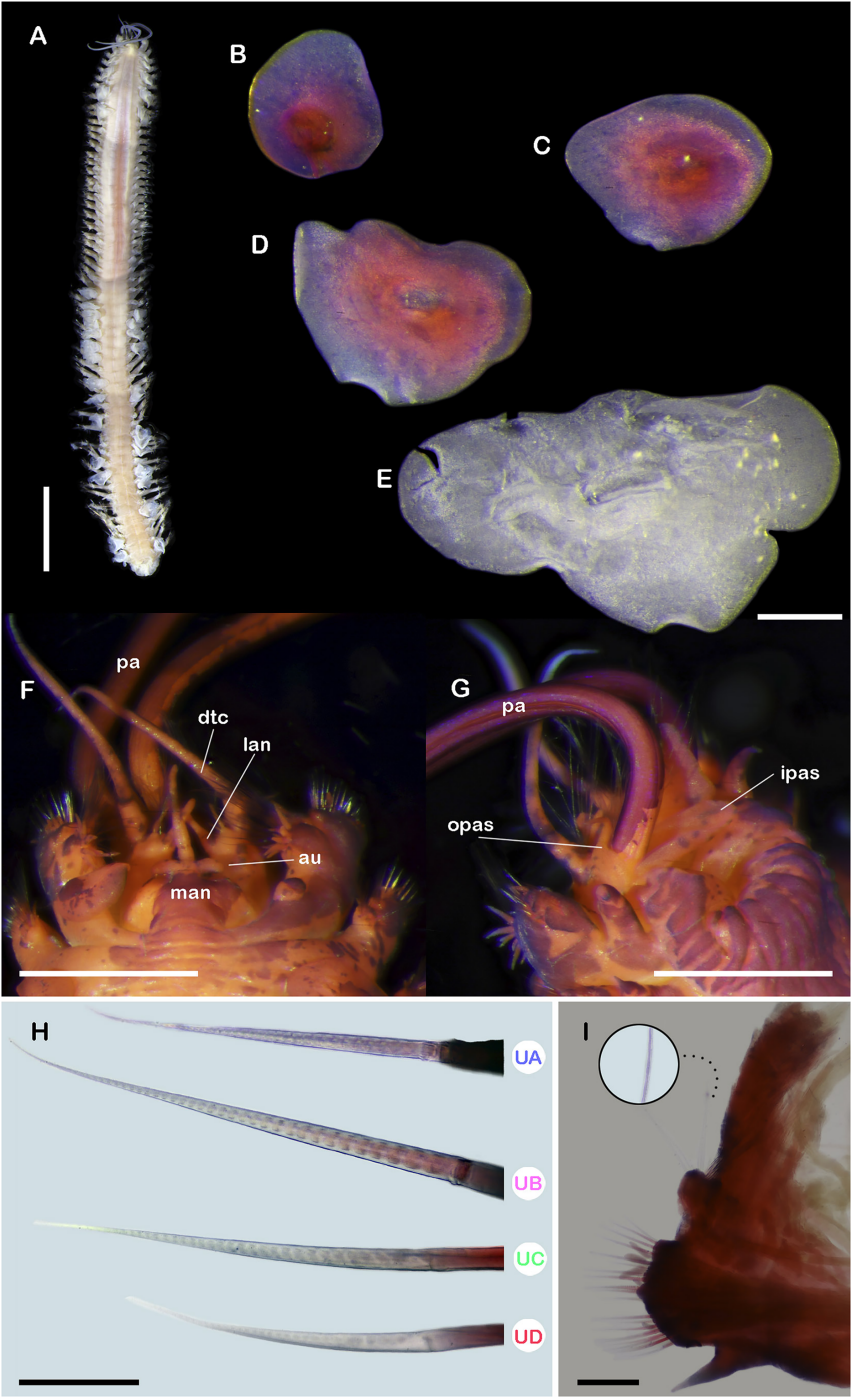
*Ehlersileanira longistyla* n. sp., holotype (MZB.Pol. 00343). (A) Dorsal view. (B) First left elytron. (C) Second right elytron. (D) Median right elytron. (E) Posterior right elytron. (F) Anterior end, dorsal view. (G) Anterior end, ventral view. (H) Neurochaetae from segment 3. (I) Parapodium from segment 3, anterior view, inset: close-up of notochaetae. Abbreviations: au, auricles; dtc, dorsal tentacular cirri; ipas, inner palpal sheath; lan, lateral antenna; man, median antenna; opas, outer palpal sheath; pa, palp; UA, upper group; UB, upper middle group; UC, middle group; UD, lowest group. Scale bars: A, 5 mm; F,G, 1 mm; B, C, D, E, 500 µm; I, 200 µm; H, 50 µm.

**Figure 4:**
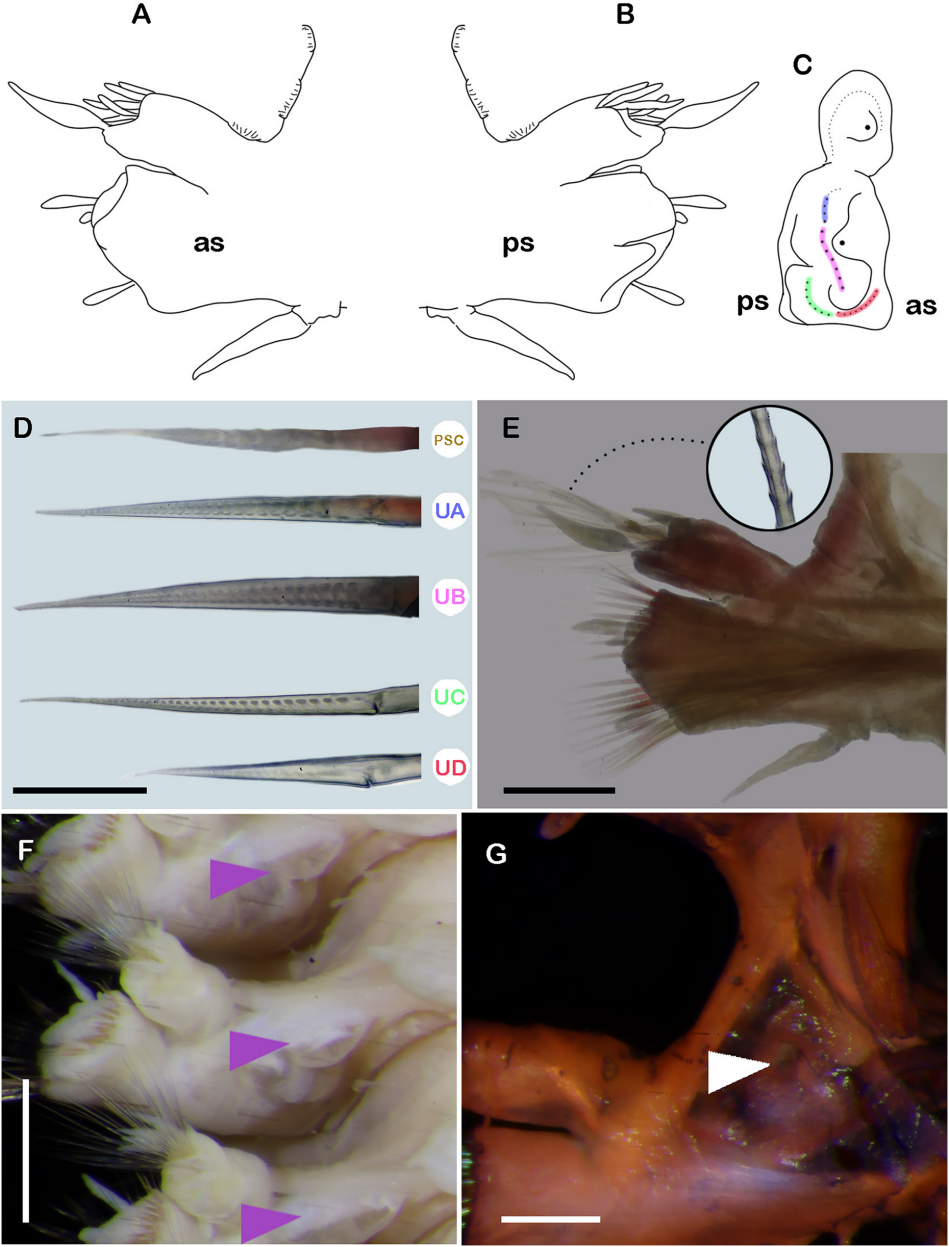
*Ehlersileanira longistyla* n. sp., holotype (MZB.Pol. 00343). (A) Drawing of parapodium from segment 19, anterior view. (B) Drawing of same, posterior view. (C) Diagram of same, lateral view, color lines indicate insertion of neurochaetal groups, color scheme following that shown in D. (E) Parapodium from segment 19, anterior view, inset: close-up of notochaetae. (F) Right parapodia from the middle region, arrowheads indicate parapodial pore (ZRC.ANN.2184). (G) Close-up of the same. Abbreviations: PSC, Pinnate simple chaetae; UA, upper group; UB, upper middle group; UC, middle group; UD, lowest group; as, anterior side; ps, posterior side. Scale bars: E, F, 200 µm; D, G, 50 µm.

**Figure 5:**
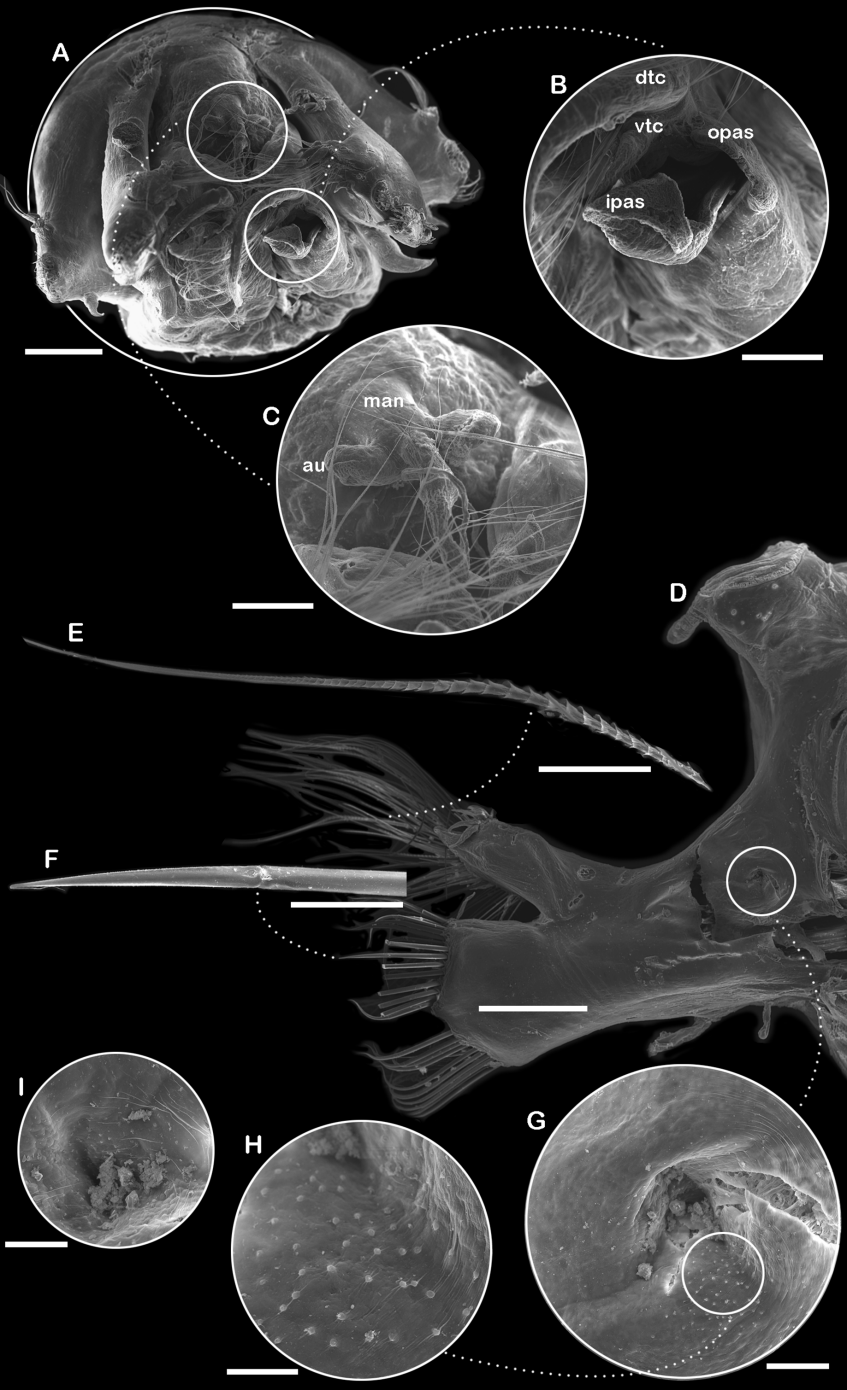
SEM micrographs of *Ehlersileanira longistyla* n. sp., paratype (ECOSUR-316). (A) Anterior end, frontal view. (B) Palpal sheath (palp removed). (C) Median antenna, frontal view. (D) Parapodium from segment 55, anterior view. (E) Notochaetae. (F) Unit B neurochaetae. (G) Parapodial pore. (H) Close-up of micro papillae. (I) Parapodial pore from segment 56. dtc, dorsal tentacular cirri; vtc, ventral tentacular cirri; opas, outer palpal sheath; ipas, inner palpal sheath; man, median antenna; au, auricles. Scale bars: A, D, 500 µm; B, C, 200 µm; E, F, 100 µm; G, I, 50 µm; H, 20 µm.

urn:lsid:zoobank.org:act:urn:lsid:zoobank.org:act:9DD8BA3D-27D8-411D-9132-036BCF50C255

*Ehlersileanira incisa*.—Chuar, Hadiyanto & Lee, 2021: 67 (*non* Grube, 1877)

**Type locality:** Off Cikiruhwetan, Indonesia.

**Type material. Holotype. MZB.Pol.00343 (sorted in a different vial)** (28 March 2018, 6° 58.624′ S 105° 53.745′ E, 481–557 m, South Java Deep-Sea Biodiversity Expedition). 1 spec. incomplete, anterior section. **Paratype. ECOSUR-316 (sorted in a different vial, partially used for SEM) (same data as holotype).** 1 spec. incomplete, anterior section in poor condition. **Paratypes. ZRC.ANN.2185** (2 spec. one with pores) (29 March 2018, 7° 42.912′ S 107° 36.559′ E, 312–525 m, South Java Deep-Sea Biodiversity Expedition); **Paratype. ZRC.ANN.2399** (1 spec. with pores) (28 March 2018, 6° 57.221′ S 105° 54.754′ E, 517–727m, South Java Deep-Sea Biodiversity Expedition). **Paratype. ZRC.ANN.2184 (**1 spec. two fragments) (29 March 2018, 7° 42.912′ S 107° 36.559′ E, 312–525 m, South Java Deep-Sea Biodiversity Expedition). **Paratype. ZRC.ANN**.**2186** (1 spec.) (27 March 2018, 6° 46.458′ S 105° 07.068′ E, 864–870 m, South Java Deep-Sea Biodiversity Expedition). **Paratype. ZRC**.**ANN.2183** (1 spec, damaged with pharynx exposed, with an anterior fragment of Polynoidae) (01 April 2018, 7° 51.120′ S 107° 46.245′ E, 637–689 m, South Java Deep-Sea Biodiversity Expedition).

**Description.** Holotype (**MZB.Pol**.**00343**) incomplete with 52 segments, 60 mm long, 23 mm to segment 30, 5 mm wide. Body slender, cylindrical, slightly wider in anterior section, damaged posteriorly; pale orange and slightly bright orange due to the muscular pharynx (Fig. 3A). Mid-dorsal line smooth, elytra present in anterior segments, a few remnants in posterior region, venter smooth. Elytrophores on segments 2, 4, 5, 7, then alternate segment to 25, then present in all segments. Elytrophores bulbous, stout, larger in posterior segments.

Prostomium pale orange, whitish on cerebral lobes; oval, wider than long. Eyes lacking. Lateral antennae long, as long as parapodium, inserted on inner dorsal side of tentacular segment. Median antenna with ceratophore long, as long as prostomial length, style biarticulate, proximal style long, 2× distal style length; inserted on the anterior margin of the prostomium. Auricles spatulate, large, as long as ceratophore (Figs. 3F, 5C). Nuchal organs small, inserted occipitally. Tentacular segment uniramous, chaetae verticillate, stylodes few, 1–2 dorsal lanceolate stylodes. Dorsal tentacular cirri long, 5× longer than tentacular neuropodia, ventral tentacular cirri short, as long as tentacular neuropodia. Facial tubercle barely visible between palps. Palps long, reaching segment 17. Inner palpal sheaths long, outer palpal sheaths short, ¼ shorter than inner palpal sheath (Figs. 3G, 5B). Buccal cirri slightly thicker and shorter than other ventral cirri. Buccal ctenidial pads, short, barely visible, inserted anterolaterally on buccal aperture. Ctenidial pads from segment 2; segment 2 with 1–2 ctenidial pads: one pad placed on dorsolateral surface on the segment; one ctenidial pad placed on dorsal side of notopodia. Dorsal pedunculate ctenidia from segment 5, inserted between rudimentary branchiae and elytrophore. Ventral intersegmental pedunculate ctenidia from segment 10. Branchiae from segment 7, becoming larger from segment 14, 2× as long as anterior ones, filiform with cilia (Fig. 3A). Tubular nephridia visible from segment 22, short, ¼ as long as ventral cirri.

All elytra with smooth surfaces and margins. First right elytron missing. First left elytron sub-quadrangular (Fig. 3B). Second right elytron rhomboid (Fig. 3C). Median elytra larger, oblong, margins sinuous (Fig. 3D). Posterior right elytra larger, proximally and laterally expanded, margins sinuous (Fig. 3E).

Segment 3 (Fig. 3I). Notopodia rounded, reduced, ½ as long as neuropodia; acicular lobe not differentiated, with no stylodes. Notacicula thick, displaced ventrally, barely protruding through the body wall. Notochaetae with up to 15 simple verticillate chaetae, smallest 5× as long as notopodia, longest 8× as long (Fig. 3I, inset). Neuropodia quadrate. Prechaetal lobe entire with two stylodes. Postchaetal lobes barely differentiated, divided by a shallow notch, with seven stylodes. Neuracicula thick, inserted in prechaetal lobe, not protruding from the body wall. Neurochaetae only spinigers, handles smooth, slender (Fig. 3H). Upper group (Unit A) with three chaetae, blades long, 16× as long as they are wide. Upper middle group (unit B) with six chaetae, blades long, 22× as long as they are wide. Middle group (unit C), four chaetae, blades medium-sized, 15× as long as they are wide. Lowest group (unit D) with four chaetae, blades medium-sized, 12× as long as they are wide. Ventral cirri slender, as long as neuropodia.

Segment 19 (middle segment) (Figs. 4A, 4B, 4C, 4E): Notopodia rectangular, slightly longer than neuropodia; acicular lobe differentiated, enlarged with two long subdistal dendritic stylodes. Notacicula thick, not protruding from the body wall. Notochaetae with up to 20 simple verticillate chaetae, smallest barely longer than notopodia, longest 2× longer than notopodia (Figs. 4E, inset; 5E). Neuropodia conical. Prechaetal lobe not differentiated, with no stylodes. Postchaetal lobes well differentiated, with three stylodes. Neuracicula thick, inserted in prechaetal lobe, barely protruding from body wall. Neurochaetae three pinnate simple chaetae and spinigers, handles smooth, slender (Fig. 4D). Upper group (unit A) with four chaetae, blades short or medium-sized, up to 11–13× as long as they are wide. Upper middle group (unit B) with five chaetae, blades short, 9× as long as they are wide. Middle group (unit C) with six chaetae, blades medium sized, 15× as long as they are wide. Lowest group (unit D) with four chaetae, blades short, 8× as long as they are wide. Ventral cirri shorter than parapodia (Figs. 4A, 4B, 4E, 5F).

Posterior region lost.

**Variation.** Paratypes include three more specimens, all of them incomplete. TL = 62–86.5 mm, TL30 = 20–25 mm, TW = 6–8.5 mm.

An interramal pore was noted in some specimens (paratype, **ZRC.ANN.2184**; non-types, **ZRC.ANN.2399**; **ZRC.ANN.2185**) (Figs. 4F, 4G, 5G, 5I). The pore is in the anterior surface of parapodia, next to notopodia, and it is extended from segment 6 to at least segment 52. Unfortunately, the specimens are incomplete; therefore, the extent of the pores’ distribution cannot be determined with certainty. Below the parapodial pore, small papillae were observed at the entrance (Fig. 5H). Tubular nephridia starts at segment 23 (**ZRC.ANN.2184**), or 24 (**ECOSUR-316**).

**Remarks.** *Ehlersileanira longistyla* n. sp. is described based on mature specimens from Indonesia formerly identified as *E. incisa* by Chuar, Hadiyanto & Lee (2021). In the region, *E. longistyla* n. sp. can be confused with *E. vulturis, E. andamanensis* and *E. marinduquensis* n. sp. However, these species differ from *E. longistyla* n. sp. in having the median antennal style small, as long as the ceratophore, and inner palpal sheath smaller, subequal to the outer palpal sheath (Aungtonya & Eibye-Jacobsen, 2016: 112, fig. 1E).

Another relevant difference between *E. longistyla* n. sp. and *E. andamanensis* is that the latter possesses ctenidial pads below the auricles and elytra from middle segments are rather quadrate (Aungtonya & Eibye-Jacobsen, 2016: 113, fig. 2H), whereas *E. longistyla* n. sp. presents only auricles and more complex elytra with sinuous margins.

*Ehlersileanira longistyla* n. sp. also differs from the rest of *Ehlersileanira* species in having a long median antennal style, a large inner palpal sheath, elytra with sinuous margins, neurochaetal unit D with non-canaliculate blades, and an interramal pore in the parapodia from posterior segments.

**Etymology.** The species name *longistyla* is a combination of the Latin adjective *longus* (-*a*, -*um*) meaning long, and *stylus* (-*i*, -*um*) meaning column or pillar. The name indicates the long median antennal style of the species, and it is feminine to agree with the gender of the genus (ICZN 1999, Art. 31.2).

***Ehlersileanira marinduquensis* n. sp.**

Figures 6–8

**Figure 6:**
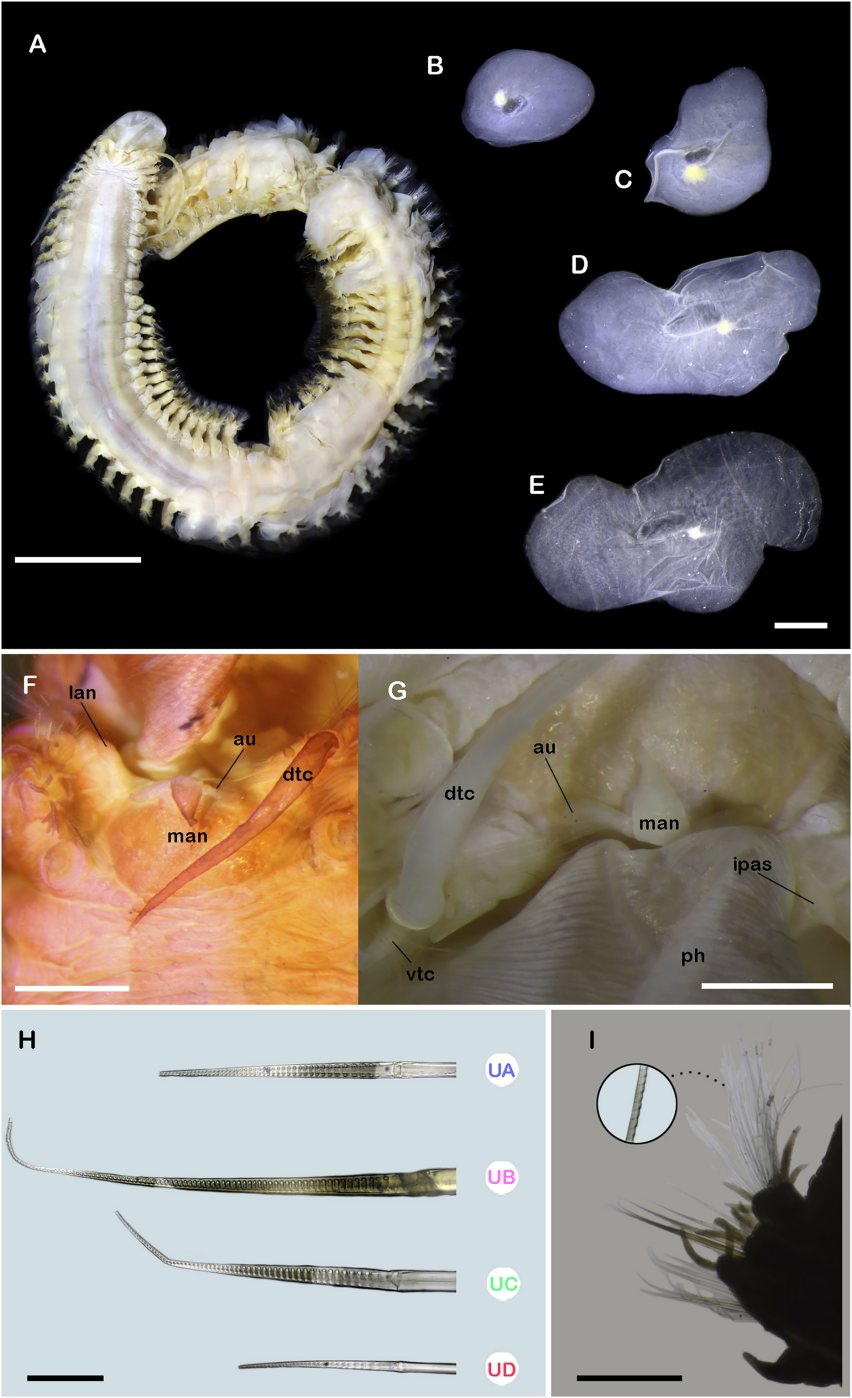
*Ehlersileanira marinduquensis* n. sp., holotype (USNM 17516). (A) Dorsal view. (B) First left elytron. (C) Second right elytron. (D) Median right elytron. (E) Posterior right elytron. (F) Anterior end, dorsal view. (G) Anterior end frontal view. (H) Neurochaetae from segment 3. (I) Parapodium from segment 3, anterior view, inset: close-up of notochaetae. Abbreviations: au, auricles; dtc, dorsal tentacular cirri; ipas, inner palpal sheath; lan, lateral antenna; man, median antenna; opas, outer palpal sheath; pa, palp; UA, upper group; UB, upper middle group; UC, middle group; UD, lowest group; vtc, ventral tentacular cirri; ph, pharynx. Scale bars: A, 10 mm; B, C, D, E, F, 1 mm; G, I, 500 µm; H, 50 µm.

**Figure 7:**
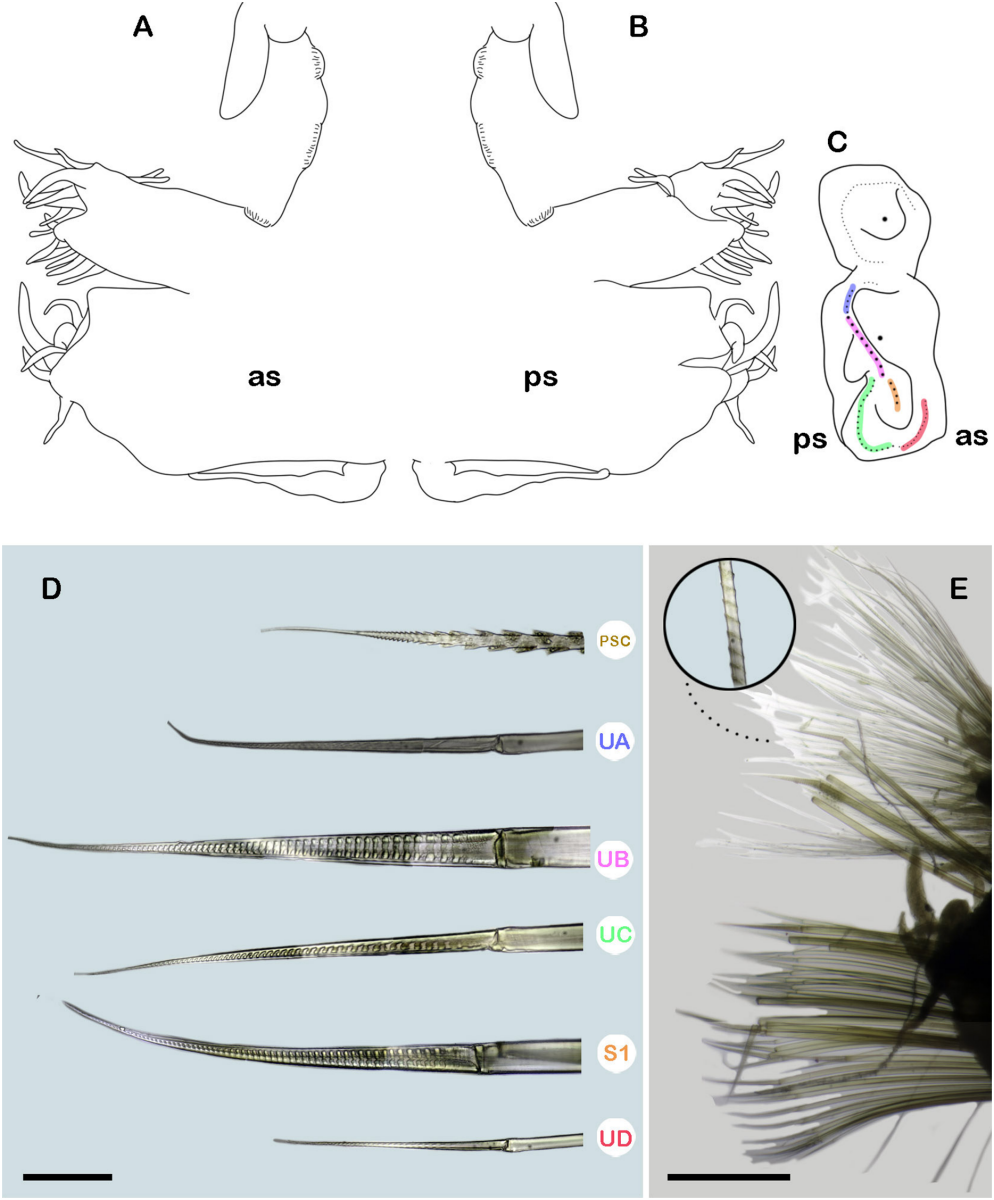
*Ehlersileanira marinduquensis* n. sp., holotype (USNM 17516). (A) Drawing of parapodium from segment 20, anterior view. (B) Drawing of same, posterior view. (C) Diagram of same, lateral view, color lines indicate insertion of neurochaetal groups, color scheme following that shown in D. (E) Parapodium from segment 20, anterior view, inset: close-up of notochaetae. Abbreviations: PSC, Pinnate simple chaetae; S1, lower middle group; UA, upper group; UB, upper middle group; UC, middle group; UD, lowest group; as, anterior side; ps, posterior side. Scale bars: E, 500 µm; D, 50 µm.

**Figure 8:**
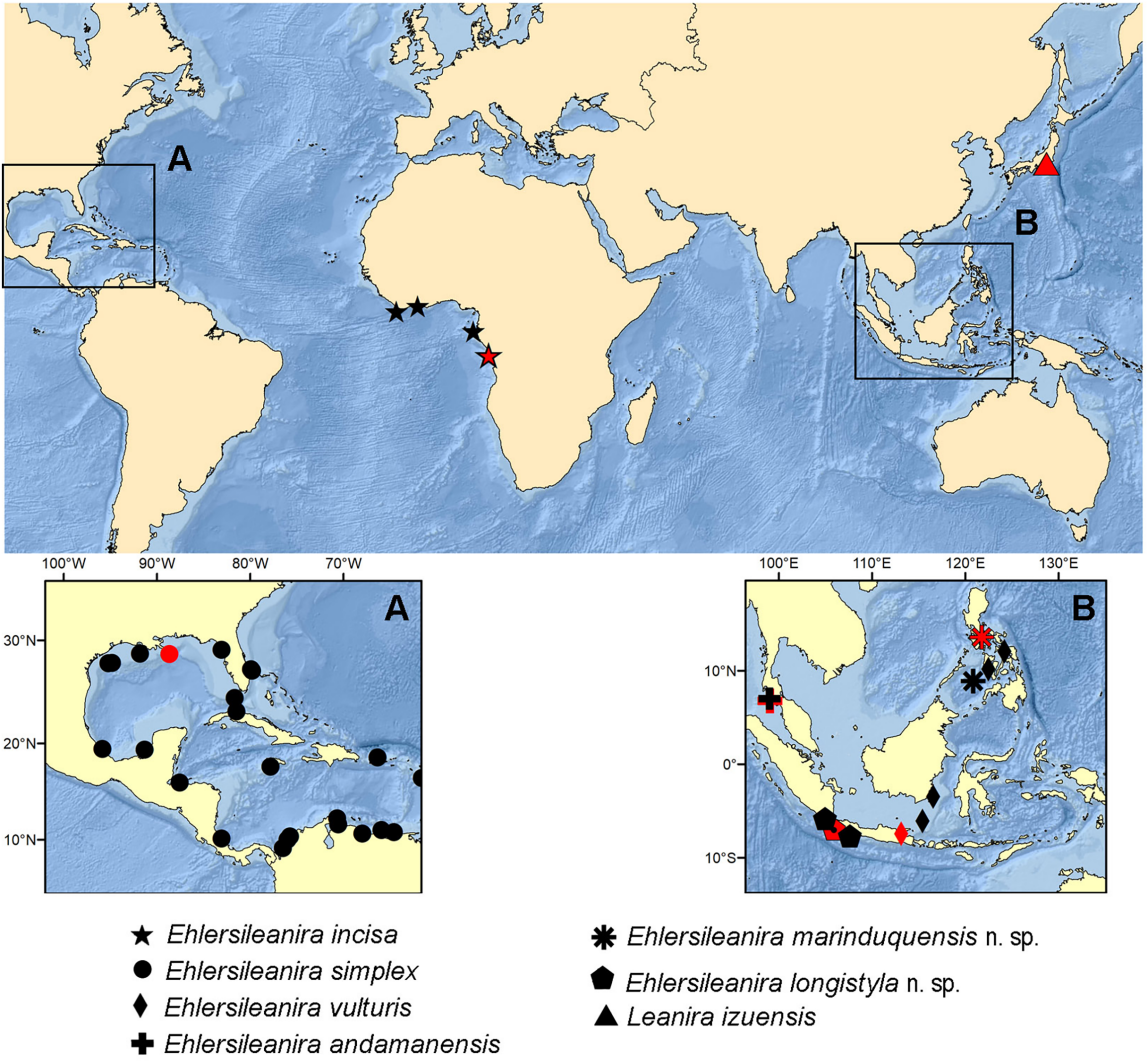
Map of the distribution of *Ehlersileanira* and related species worldwide. (A) Gulf of Mexico and Caribbean Sea. (B) Malay Archipelago. Red symbols denote the type locality of the species. Black symbols denote the historic records of the species. © 2025 General Bathymetric Chart of the Oceans (GEBCO).

urn:lsid:zoobank.org:act:urn:lsid:zoobank.org:act:3E5F6813-BCFD-48E0-8FA5-27D079D8F518

*Ehlersileanira incisa*.—Pettibone, 1970b: 19. Syn (partim *non* Grube, 1877).

**Type locality:** Marinduque Island, the Philippines.

**Type material. Holotype. USNM 17516.** 1 spec. Marinduque Island & vicinity, Sta. 5375, 195.7 m, 2 March 1909, USS Albatross.

**Paratype. USNM 17514.** 1 spec. Jolo Sea, P.I., Sta. 5,423, 929 m, 31 March 1909, P.I. USS Albatross.

**Description.** Holotype (**USNM 17516**) incomplete with 62 segments, 73 mm long, 23 mm to segment 30, 8 mm wide. Body thick, cylindrical, slightly narrower in the anterior section, damaged posteriorly; pale yellow, and slightly whitish due to the muscular pharynx (Fig. 6A). Mid-dorsal line smooth, elytra present in anterior and posterior segments, venter smooth. Elytrophores on segments 2, 4, 5, 7, then alternate segment to 25, then present in all segments. Elytrophores bulbous, stout, fleshy, larger in posterior segment.

Prostomium pale yellow, bright yellow on cerebral lobes; oval, wider than long. Eyes lacking. Lateral antennae short, ¼ as long as parapodium, inserted on inner dorsal side of tentacular segment. Median antenna with ceratophore short, ½ as long as prostomial length, style biarticulate, proximal style long, 4× distal style length; inserted on the anterior prostomial margin. Auricles spatulate, large, as long as ceratophore. Nuchal organs small, inserted occipitally. Tentacular segment uniramous, chaetae verticillate, stylodes few, 5–6 dorsal dendritic stylodes. Dorsal tentacular cirri long, 5× longer than tentacular neuropodia, ventral tentacular cirri short, barely shorter than tentacular neuropodia. Facial tubercle barely visible between palps. Palps long, reaching segment 14. Inner palpal sheaths short, outer palpal sheath shorter, ½ as large as inner palpal sheath. Buccal cirri slightly thicker and shorter than the remaining ventral cirri. Buccal ctenidial pads not seen. Ctenidial pads from segment 2; segment 2 with 1–2 ctenidial pads: one pad placed on dorsolateral surface on the segment; one ctenidial pad placed on dorsal side of the notopodia. Dorsal pedunculate ctenidia from segment 7, inserted between branchiae and elytrophore. Ventral intersegmental pedunculate ctenidia from segment 11. Branchiae from segment 9, becoming larger from segment 27, 2× as long as the anterior ones, filiform with cilia (Fig. 6A). Tubular nephridia from segment 18, short, as long as ventral cirri. In posterior segments, tubular nephridia bifurcate.

All elytra with smooth surfaces and margins. First right elytron oval (Fig. 6B). Second right elytron oblong, margins sinuous (Fig. 6C). Median elytron larger, oblong, proximally and laterally slightly expanded, margins sinuous (Fig. 6D). Posterior elytra larger, and proximally expanded, curved, laterally and posteriorly expanded, margins sinuous (Fig. 6E).

Segment 3 (Fig. 6F). Notopodia rounded, as long as neuropodia; acicular lobe not differentiated with four stylodes. Notacicula thick, not protruding from body wall. Notochaetae with up to 40 simple verticillate chaetae, smallest as long as notopodia, longest 5× as long (Fig. 6I, inset). Neuropodia truncate. Prechaetal lobe entire with no stylodes. Postchaetal lobes barely differentiated, divided by a shallow notch, with two stylodes. Neuracicula thick, inserted in prechaetal lobe, not protruding from body wall. Neurochaetae only spinigers, handles smooth, slender (Fig. 6H). Upper group (Unit A) with two chaetae, blades medium-sized, 14× as long as they are wide. Upper middle group (unit B) with four chaetae, blades medium-sized, 16× as long as they are wide. Middle group (unit C) with six chaetae, blades medium-sized, 14–16× as long as they are wide. Lowest group (unit D) with five chaetae, blades long, 22× as long as they are wide. Ventral cirri thick, slightly shorter than neuropodia.

Segment 20 (middle segment) (Figs. 7A, 7B, 7C, 7E). Notopodia rectangular, with up to 15 distal and subdistal dendritic stylodes. Notacicula thick, not protruding from the body wall. Notochaetae with up to 80 simple verticillate chaetae, smallest as long as notopodia, longest barely longer than notopodia (Fig. 7E, inset). Neuropodia conical. Prechaetal lobe entire, with 2 dendritic stylodes on the lateral external margin. Postchaetal lobes well differentiated with a deep notch, with three stylodes. Neuracicula thick, inserted in prechaetal lobe, not protruding from body wall. Neurochaetae 4 pinnate simple chaetae and spinigers, handles slender, smooth (Fig. 7D). Upper group (unit A) with two chaetae, blades long, 19–20× as long as they are wide. Upper middle group (unit B) with four chaetae, blades long, 10× as long as they are wide. Middle group (unit C) with nine chaetae, blades long, 21–23× as long as they are wide. Lower group (subunit 1) with two chaetae, blades medium-sized, 15× as long as they are wide. Lowest group (unit D) with seven chaetae, blades long, 27× as long as they are wide. Ventral cirri shorter than parapodia (Figs. 7A, 7B).

Posterior region lost.

**Remarks.** *Ehlersileanira marinduquensis* n. sp. is described based on two specimens from the Philippines; it resembles *E. vulturis* by having a short style in median antenna, sinuous elytral margins and small auricles. The two species differ, however, in terms of the shape of the elytra, the size of the dorsal tentacular cirri and palps, the body shape, and the size of the neurochaetal units blades.

In *E. marinduquensis* n. sp. the elytra from the middle region have proximal and lateral margins expanded and rounded, whereas those in *E. vulturis* not expanded and are narrower and slightly rounded, respectively. Regarding the anterior appendages, in *E. marinduquensis* n. sp. the dorsal tentacular cirri are 5× longer than tentacular parapodia and palps reach segment 14; whereas *E. vulturis* has longer appendages which are up to 6× longer than the tentacular parapodia and palps reach segment 23.

The neurochaetae are also different between these species, with *E. marinduquensis* n. sp. having a smaller variety of units, and relatively longer blades. In *E. vulturis* there are two more types of units, such as subunit 1 and 2, and shorter blades. For instance, unit B chaetae in *E. marinduquensis* n. sp. are thick, with a long blade, while the same unit in *E. vulturis* is slimmer and shorter.

**Etymology.**

The specific epithet is an adjective based on the type locality, which is Marinduque Island, the Philippines.

**Key to species of *Ehlersileanira* Pettibone, 1970b (modified from Cruz-Gómez 2022)**
1 Median antennal ceratophore with auricles only2
- Median antennal ceratophore with auricles and ctenidia*E. andamanensis* Aungtonya & Eibye-Jacobsen, 2016, Thailand, Central Indo-Pacific.
2(1) Median antennal style short, as long as the ceratophore; auricles small, ½ as long as ceratophore length (Fig. 1E)3
- Median antennal style long, longer than the ceratophore; auricles large, as long as ceratophore length (Fig. 3F)6
3(2) Branchiae from at least segment 16*E. simplex* (Ehlers, 1887), off Louisiana, Gulf of Mexico
- Branchiae from at least segment 74
4(3) Auricles reniform (Fig. 1E); palps long, reaching segment 23*E. vulturis* (Horst, 1917), Indonesia, Central Indo-Pacific.
- Auricles spatulate (Fig. 6F); palps short, reaching at least segment 135
5(4) Body slender; elytra from the middle region with distal margin reduced with angle*E. incisa* (Grube, 1877), Congo, Western Africa.
- Body thick; elytra from the middle region with distal margin expanded and rounded (Figs. 6D, 6E)*E. marinduquensis* n. sp., the Philippines, Central Indo-Pacific.
6(2) Tentacular cirri short, about 2× tentacular parapodia; palps reaching segment 14; branchiae from segment 15; elytra from the middle region with expanded margins; interramal pore absent*Leanira izuensis* Takahashi, 1938, Izu Peninsula, Japan^1^ 1Currently, *L. izuensis* is under the combination of *Sthenolepis* Willey, 1905, proposed by Imajima & Hartman (1964), supported by the diagnosis of the species. However, the morphology described by Takahashi (1938) agrees with *Ehlersileanira*; we suggest revisiting the type specimen before making any formal nomenclatural act..
- Tentacular cirri long, about 5× tentacular parapodia; palps reaching segment 17; branchiae from segment 7; elytra from the middle region without expanded margins (Figs. 3D, 3E); interramal pore might be present (Figs. 4F, 4G, 5G–5I)*E. longistyla* n. sp., Indonesia, Central Indo-Pacific.

## Discussion

Pettibone (1970b, 1970c) revised the genus *Leanira* and proposed a series of genera to include species that do not fit the diagnosis of *Leanira*, such as *Horstileanira* Pettibone, 1970, *Neoleanira* Pettibone, 1970, and *Ehlersileanira*. Regarding *Ehlersileanira*, she classified four species as junior synonyms of *Sthenelais incisa*, the type species of *Ehlersileanira* described from Congo, Africa. Recently, a closer examination of the morphology of specimens identified as its junior and senior synonyms has shown that these species are, in fact, distinct (Cruz-Gómez, 2022b; this study).

In the analysis that led to these taxonomic decisions, the morphology considered included appendages from the anterior and middle regions, such as the parapodia and elytra. The same features were also examined by Pettibone (1970b) in her revision; however, some details were overlooked. The size of the style in the median antenna, the shape of the auricles and elytra, neurochaetal blades, branchia, ctenidial pads, and tubular nephridia were features where differences within the species were observed. The differences between *E. incisa* and its evaluated junior synonyms were subtle but consistent (see Cruz-Gómez, 2022a; see remarks of *E. vulturis*).

As we considered the morphology previously listed here, some new characters became apparent. Specimens of *Ehlersileanira longistyla* n. sp. exhibit a parapodial pore in the posterior segments; this structure has not been previously recorded in the family Sigalionidae. A detailed investigation of this structure is beyond the scope of this study. Parapodial pores have been recorded in other Annelida families like Syllidae (*i.e*., Exogoninae), where they are mostly related to glandular functions (Aguado & San Martín, 2009). On *Ehlersileanira longistyla* n. sp., masses of what looked like sperm, were observed near the entrance of the pore (Figs. 5G–5I).

Other structures that were also considered in the description and delimitation of *Ehlersileanira* included the segment from where the branchiae, ctenidial pads, and tubular nephridia begin to occur, as well as the presence of secondary tubular nephridia. The recognition of the segments where easily detachable structures, such as ctenidial pads or tubular nephridia are inserted can be challenging, as are the scars left behind which are sometimes impossible to detect. However, considering this, the definition of the species not only covers those features (see Key to species). Studies on the taxonomic value of branchiae have been heterogeneous. Grube (1877) recognized the change in the size of the branchia through the body, and posterior descriptions pointed out the presence of rudimentary branchiae in the anterior segments. Horst (1917) observed the same pattern in *E. vulturis*, but he specified the site of insertion of the branchiae to be lateral to the elytrophore. Takahashi (1938) did not mention any rudimentary branchiae but described the segment where the branchiae associated with the elytrophore begin to occur. Subsequent contributions observed the presence of rudimentary branchiae in anterior segments (Pettibone, 1970b; Aungtonya & Eibye-Jacobsen, 2016; Cruz-Gómez, 2022a) and where the proper branchiae appear in posterior segments. Here, we follow the same approach; however, we also considered the size of the branchiae (see Materials and Methods section).

Until recently, *Ehlersileanira* was considered a monotypic genus, with only *E. incisa* as the unique representative, including its junior synonyms. Two junior synonyms are now reinstated, namely *E. simplex* and *E. vulturis* (Cruz-Gómez, 2022a; this study), leaving one junior synonym for future re-evaluation (*i.e*., *L. izuensis* from Japan).

The family Sigalionidae is found worldwide at different depths and marine habitats but is more common in sandy bottoms of tropical regions (Gonzalez et al., 2018; Eibye-Jacobsen, Aungtonya & Gonzalez, 2022). *Ehlersileanira* follows this pattern by being present in tropical and subtropical areas, with the highest diversity in the Indo-Pacific region (Fig. 8). Members of *Ehlersileanira* have a wide bathymetric distribution ranging from 30 to 929 m in depth, being more present in the first layer of water in the sublittoral zone. *Ehlersileanira incisa* and *E. andamanensis* can be found only in this zone, while *E. simplex*, *E. vulturis*, and *E. marinduquensis* n. sp. with a wider range of bathymetric distribution, can also be found here. In deeper water, beyond 200 m, we can find *E. longistyla* n. sp. reaching 870 m depth. Also, *E. simplex* and *E. marinduquensis* n. sp. are found in this area, the latter with the widest range of depth, 30 to 710 m, and the deepest record of the genus with 929 m.

It is not new that the region is considered a global marine biodiversity hotspot; however, regarding polychaetes, the lack of information is notorious if it is contrasted to other groups of invertebrates (Ybañez & Aspe, 2024). This contribution amplifies the knowledge of endemic species in the region with the addition of two new species. Interestingly, polychaetes are poorly studied in this marine hotspot (Pamungkas & Glasby, 2019; Ybañez & Aspe, 2024). According to Pamungkas & Glasby (2019) and Siallagan et al. (2023), the main limitation to studying polychaetes in the region is the lack of taxonomists, aggravated by the lack of funding for taxonomical revisions.

## Conclusions

*Ehlersileanira* is a genus rarely found and, therefore, poorly studied. The complex morphology and incomplete descriptions in classical contributions have made its taxonomy challenging. However, the group is more diverse than previously thought. Prior to this study, only two species had been recorded from the central Indo-Pacific region. This contribution adds two more, both representing new species. In addition, the type material of *L. vulturis* was revisited, and after close examination, it was reinstated as *E. vulturis* and differentiated from *E. incisa*. Together with the two new species, *Ehlersileanira longistyla* n. sp. and *Ehlersileanira marinduquensis* n. sp. the number of species in the genus has increased to six. In this study, the diagnosis of the genus *Ehlersileanira* has been extended to include the presence of parapodial pores in posterior segments. The description was complemented with SEM imaging as well as photographs. In the description of the neurochaetae, the use of the unit terminology for neurochaetae was also implemented. The identification key for the genus included in this study will assist in future taxonomical and ecological studies of the Sigalionidae in the Indo-Pacific region.

## Supplemental Information

10.7717/peerj.20886/supp-1 Supplemental Information 1 Raw data.

## Additional Information and Declarations

### Field Study Permissions

The following information was supplied relating to field study approvals (*i.e*., approving body and any reference numbers):

Ministry of Research, Technology, & Higher Education, Indonesia

### Data Availability

The following information was supplied regarding data availability:

The raw data is available in the Supplemental File.

### New Species Registration

The following information was supplied regarding the registration of a newly described species:

Ehlersileanira longistyla LSID: urn:lsid:zoobank.org:act:urn:lsid:zoobank.org:act:9DD8BA3D-27D8-411D-9132-036BCF50C255

Ehlersileanira marinduquensis LSID: urn:lsid:zoobank.org:act:urn:lsid:zoobank.org:act:3E5F6813-BCFD-48E0-8FA5-27D079D8F518

Publication LSID: urn:lsid:zoobank.org:pub:517D778D-ED68-4DFA-A708-FB57FFDDDADF.
